# Barriers to dog bite case management and dog rabies vaccination in Soroti district, Uganda

**DOI:** 10.3389/fvets.2026.1814100

**Published:** 2026-05-27

**Authors:** Terence Odoch, Doreen Agado, Salome Dürr, Samuel George Okech, Juliet Kiguli, Clovice Kankya, Dickson Akankwatsa, Sonja Hartnack

**Affiliations:** 1 Department of Biosecurity, Ecosystems and Veterinary Public Health, School of Biosecurity, Biotechnical and Laboratory Sciences, College of Veterinary Medicine, Animal Resources and Biosecurity, Makerere University, Kampala, Uganda; 2 Veterinary Public Health Institute, Vetsuisse Faculty, University of Bern, Bern, Switzerland; 3 Department of Veterinary Pharmacy, Clinical and Comparative Medicine, School of Veterinary Medicine and Animal Resources, Makerere University, Kampala, Uganda; 4 Graduate School for Cellular and Biomedical Sciences, University of Bern, Bern, Switzerland; 5 Department of Community Health and Behavioral sciences, School of Public Health, College of Health Sciences, Makerere University, Kampala, Uganda; 6 Department of Epidemiology and Biostatistics, School of Public Health, College of Health Sciences, Makerere University, Kampala, Uganda; 7 Department of Global Health Security, Infectious Disease Institute, Makerere University, Kampala, Uganda; 8 Section of Epidemiology, Vetsuisse Faculty, University of Zurich, Zurich, Switzerland; 9 Clinical Department for Livestock and Transformation of Food Systems Centre for Veterinary Public Health and One Health, University of Veterinary Medicine, Vienna, Austria

**Keywords:** barriers, mass canine vaccination, one health, rabies, rabies control, socio-ecological model

## Abstract

**Introduction:**

Rabies, a neglected tropical disease, causes about 59,000 annual human fatalities globally, the majority of which result from dog bites. Although rabies is preventable by vaccinating dogs and providing post-exposure prophylaxis (PEP) to humans, dog vaccination coverage in Uganda remains low at approximately 10% and not all human dog bite cases receive PEP. This study sought to understand barriers to dog bite case management and dog rabies vaccination.

**Methods:**

This study, conducted between September 2022 and March 2023, involved 10 focus group discussions (FGDs) with dog owners and eight key informant interviews (KIIs) with opinion leaders, veterinary and health professionals. Participants were purposively selected based on their experience with dog vaccination and bite case management. Data was collected from Soroti Municipality (peri-urban) and Kamuda sub-county (rural) using structured interview guides premised on the socio-ecological model. Transcripts were coded and analyzed thematically using NVivo software (version 12.0).

**Results:**

At the individual level, limited first aid knowledge, few available PEP doses and high PEP costs were major barriers to proper dog bite case management. Community level barriers included poor management of roaming dogs and insufficient knowledge on managing biting dogs. Organizational barriers included frequent PEP stock-outs, improper cold-chain system in health facilities and poor communication between relevant health sectors. Regarding dog rabies vaccination, individual level barriers were, uncertainty about vaccination frequency, high vaccine costs and irresponsible dog ownership. Community level barriers centered on inadequate communication about dog vaccination campaigns and poor communication between the dog owners and veterinary officers, while organizational barriers included inaccurate data on dog population, inadequate staffing and logistical constraints.

**Discussion:**

The study’s findings revealed that effective rabies prevention is constrained by interrelated individual, community and systemic factors. To address these gaps, district health authorities should provide continuous community education on dog bite first aid and timely seeking of care, ensure consistent availability of PEP through improved supply chain management, and implement subsidized or free mass dog vaccination campaigns. In addition, local governments should improve dog registration systems and enforce responsible dog ownership, while strengthening coordination between human and animal health sectors through One Health approaches.

## Introduction

Rabies, classified as a Neglected Tropical Disease by the World Health Organization (WHO), poses a persistent global health threat with staggering public health consequences (1). Annually, it leads to approximately 59,000 human fatalities, causing an economic burden of approximately US$ 8.6 billion and accounting for a loss of 3.7 million disability-adjusted life years. This crisis is mainly in Asia and sub-Saharan Africa, where 95% of cases are concentrated (2). Despite successful rabies elimination interventions in western countries, sub-Saharan African nations, including Uganda, continue to grapple with substantial morbidity and mortality due to inadequate monitoring, prevention, and control measures (2, 3).

In Uganda, rabies remains endemic, particularly in areas of high transmission risk, where 89–95% of the population resides (3). The reservoirs of this zoonotic disease include mammals like dogs, raccoons, bats, and other wildlife, with over 90% of human rabies cases arising from bites inflicted by rabid domestic dogs (4). Dogs are deeply ingrained in Ugandan culture for various purposes such as hunting, companionship, and security, thus roam freely among communities, contributing to the challenges of dog bite management and rabies control (5–7). Soroti district, situated within the cattle keeping zones of the country, witnesses the common use of dogs for livestock protection. This contributes to the high number of free-roaming dogs and thus necessitating effective control measures for the well-being of both humans and animals.

Bites from rabid dogs serve as the primary source for rabies transmission to humans, especially in rural and peri-urban areas characterized by poor dog control and many roaming dogs (8). Recognizing the urgency of the situation, this study embarked on a comprehensive investigation into barriers to dog bite case management, and mass vaccination, as pivotal strategies for preventing and eliminating rabies in both human and animal populations (2, 9, 10). Achieving sustained participation in successive vaccination campaigns, targeting at least 70% of susceptible dogs to ensure herd immunity, is vital for the success of these strategies (2, 11).

Uganda’s Ministry of Agriculture Animal Industry and Fisheries (MAAIF) and the Ministry of Health (MOH), supported by international organizations such as WHO, Food and Agricultural Organization, World Organization for Animal Health, Global Alliance for Rabies Control and Mission Rabies, have established strategies and systems to eliminate rabies in Uganda. These efforts align with the Zero by 2030 initiative (12). Uganda’s National Rabies Elimination Strategy provides a comprehensive, multi-sectoral roadmap for rabies elimination, focusing on routine vaccination of dogs, rabies awareness, immediate wound management, surveillance, and post-exposure prophylaxis (PEP) (13). This strategy is operationalized through the Uganda One Health platform to address zoonotic threats (14).

However, despite these efforts, the effective and routine mass vaccination of dogs is rarely achieved and the country struggles with low coverage of 10%, particularly in rural communities (15). This limited progress suggests persistent and interconnected barriers operating across multiple levels of the socio-ecological model (16).

At individual level, earlier studies have reported inadequate knowledge about rabies transmission and prevention, misconceptions regarding would care, and delayed or incomplete uptake of PEP doses among bite victims (17–21). In addition, dog owners’ attitudes, previous experiences and socio-economic constraints influence their willingness to participate in vaccination campaigns (22–24).

At community level, socio-cultural beliefs, reliance on traditional healing practices, and low perceived risk of rabies contribute to delayed health-seeking behavior and inadequate compliance with recommended prevention practices (5, 25, 26). Furthermore, dog ownership practices that allow animals to roam freely, coupled with unstrict enforcement of responsible ownership, facilitate sustained transmission within communities (27, 28).

At the organizational level, studies suggest that systemic challenges such as limited access to timely and affordable PEP, vaccine stock outs, logistical constraints and limited veterinary workforce capacity may hinder effective rabies control (19, 29, 30). Weak enforcement of policies and suboptimal coordination between human and animal health sectors have also been potential constraints (14, 31).

Although previous studies have documented various barriers to rabies control, these are often in isolation, with limited integration across human and animal health systems or across socio-ecological levels (5, 17, 23, 26, 32). Moreover, there is limited context-specific evidence from high-risk settings such as Soroti district that simultaneously explores barriers to both dog bite case management and dog vaccination. Therefore, this study endeavored to identify the multifaceted challenges obstructing the widespread adoption of rabies vaccination and the effective management of dog bites in Soroti district, Uganda. To guide this analysis, the study adopted the socio-ecological model (SEM), which provides a framework for understanding how individual behavior is shaped by factors at multiple levels, including; individual, community, and organizational (16).

## Materials and methods

The methods, analysis and results sections have been written in accordance with the Consolidated Criteria for Reporting Qualitative Research (COREQ) guidelines (33).

### Study area and setting

**Figure 1:**
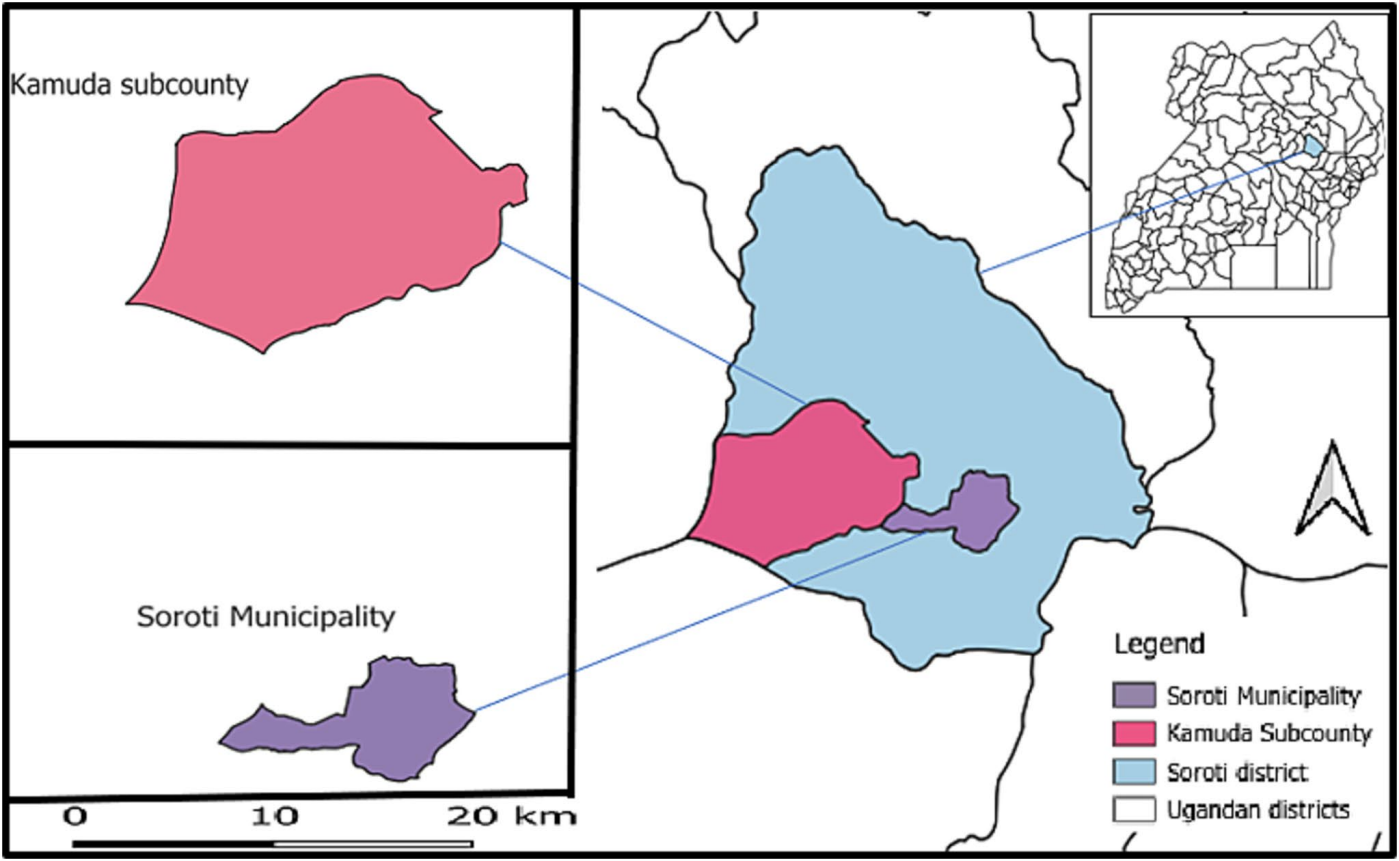
A map showing the study area on barriers to dog bite case management and dog vaccination in Soroti District, Uganda.

This study was conducted in Soroti district (Figure 1), located in the Eastern region of Uganda, specifically within the Teso sub-region. Soroti is a peri-urban district, situated approximately 290 kilometers Northeast of Kampala (Uganda’s capital city), with geographic coordinates of 1.7229^o^N, 33.5280°E. The district covers approximately 1,416 km^2^ with a human population of approximately 266,189 out of which 126,583 (47.6%) are males and 139,606 (49.2%) are females according to the population and housing census of 2024 (34). It shares borders with Katakwi district to the North, Kumi district to the East, Ngora and Serere districts to the South, Amuria district to the West. The major economic activity in this area is subsistence mixed farming with the majority being small holders. Additionally, the district lies within the major cattle keeping zones of Uganda, with livestock farmers using dogs to guard their cattle herd during day and their homes during the night. As a result, the district likely has a substantial dog population, which increases the frequency of human-dog interaction, and consequently, the risk of dog mediated rabies transmission to humans within this area.

According to the Uganda healthcare system, health service delivery is by level ranging from health centre II (HCII) to health centre four (HCIV) and then hospital level. In addition, the system embraces private ownership of health facilities to supplement the government efforts (35). The type and intensity of healthcare service is based on the level of the health facility. Soroti district has 53 health facilities (23 HCIIs, 16 HCIIIs, 2 HCIVs and 2 hospitals) (36), which provide a range of healthcare services including management of human dog bite victims.

This study was conducted between September 2022 and March 2023. The study sites were identified jointly by the research and the district local government teams that included the District Veterinary Officer and the Animal Husbandry Officer during the pre-field planning visit. Two distinct geographical settings were purposively selected, i.e., Kamuda sub-county (rural) and Soroti Municipality (peri-urban) to obtain varied insights based on setting. A total of 10 sites were randomly selected for inclusion: Sites (villages/cells) 1–6 were drawn from different parishes within Soroti Municipality, while Sites (7–10) were selected from different parishes in Kamuda sub-county.

### Study design

The study used a qualitative descriptive design to document participants’ experiences and perspectives regarding barriers to dog bite case management and dog vaccination in their own words. Through this way, the researcher maintained low level of interpretive abstraction as was used by other scholars elsewhere. The researcher’s role was to remain neutral while conducting interviews and presenting the findings, without providing meaning to participants’ views or testing a theory (37, 38).

### Participants

Participants for both key informant interviews (KIIs) and focus group discussions (FGDs) were adults drawn from the relevant institutions within Kamuda sub-county and Soroti municipality. The key informants included two village chairpersons, two religious leaders (one Muslim, one Christian), a primary school head-teacher, District Veterinary Officer, District Health Officer, and a practicing Nurse who administers PEP to dog bite victims at a selected health facility. The participants for the FGDs were either dog owners or belonged to a household that owned at least one dog.

### Sampling strategy and recruitment procedures

Purposive sampling was used to select eight participants as key informants and 96 participants for FGDs, ensuring representation across diverse perspectives relevant to dog bite management and vaccination. Participant selection included both community members and health workers so as to capture adequate insights regarding barriers to dog bite case management and dog vaccination at individual, community and organizational levels.

A total of 96 FGD participants was based on planned distribution of 10 FGDs (8–10 participants each), which is consistent with qualitative research guidelines for achieving both code and meaning saturation. Eight key informants were selected to provide expert perspectives from governance, health and veterinary sectors, which was deemed sufficient given their specialized roles.

Between September 2022 and March 2023, adult dog owners and other afore-mentioned stakeholders were invited from 10 study sites (named as Site 1–10) in two sub-counties of Soroti district. To ensure balance between rural and peri-urban perspectives, five sites (Sites 1–6) were drawn from Soroti Municipality (peri-urban) and four sites (Sites 7–10) were selected from Kamuda sub-county (rural). Selection was purposive to ensure representation across different age groups, gender and dog ownership status. Eligibility for participation in the study was limited to individuals aged 18 years and older, who were either residents of the selected villages/sites or residents within the parishes where the sites were located. Preference was given to individuals who had owned dogs for more than 1 year. The recruitment process was jointly undertaken by the research team and a community mobiliser, who had been suggested by the District Veterinary Officer for each selected site. Letters of invitation were sent to each key informant, and one researcher followed up with a phone call to confirm participation. For FGDs, after consensus regarding the participants to be involved based on eligibility, a mobiliser for each site walked to their homes to consult them regarding their participation and verbally invited those willing. A day prior to interviews, the mobiliser for each site made a phone call to remind the participants about their interviews. At the time of interviews, the mobiliser welcomed the participants and introduced the study before seeking participants’ consent. The latter was done after the research team explained all the relevant information about the study in the participants’ preferred language.

Sampling and recruitment stopped when code saturation, which was defined as a point at which no new information relevant to research question emerged from additional interviews (39). After every interview, two research assistants independently coded the transcripts, met and discussed the emerging codes, for comparison and consensus building. These emerging codes were subsequently reviewed by the senior researcher (JK) to provide any necessary guidance before conducting additional interviews. These processes continued until a code saturation point was reached. For KIIs, saturation was achieved at the 6th interview, while for FGDs, saturation was achieved at the 8th interview. Additional interviews were however performed to achieve meaning saturation (39).

### Data collection

The study applied two qualitative data collection methods: Key informant interviews (KIIs) and FGDs. Eight KIIs were conducted with opinion/community leaders as indicated under participants section, to obtain data on dog bite management and barriers to rabies vaccination programs.

The participants for the FGDs comprised of male and female dog owners (≥18 years) who either owned dogs or belonging to a household that owned at least one dog. A total of 10 FGDs were conducted, each comprising 8–10 participants. Pre-tested semi-structured and open-ended questions guided the interviews/discussions (Appendix A1), providing relevant data on; dog ownership, dog bite management and its challenges, dog vaccination and its barriers.

The convenient time for the interviews and discussions was agreed upon by the research assistants and participants so as not to inconvenience their work schedules. The venues for interviews for key informants were suggested by the interviewees themselves, to ensure convenience and privacy during the interview. For FGDs, the convenient venues were selected with the guide of community leaders and these included: school and church compounds, village public areas among others. Additionally, the discussions and interviews were conducted in the local languages (*Ateso* and *Kumam*) and/or English by two bi-lingual research assistants (DoA and TO) who received training in qualitative research. An average of 30–60 min was utilized for KIIs and between 60 and 80 min for the FGDs.

Audio recording, as well as note taking were done by the research assistants during interviews and discussions.

### Data management and analysis

All audio recordings were transcribed verbatim in *Kumam and Ateso*, then translated into English by two bi-lingual researchers (TO and DoA). To ensure translation accuracy, three transcripts were initially translated and underwent back and forth reviews and discussions to ensure consistency in wording and meaning. These were further checked by the senior researcher (JK). Any discrepancies in wording, meaning, or conceptual interpretation were discussed and harmonized, before proceeding with the remaining transcription and translation. The finalized transcripts were then uploaded into NVivo (version 12.0) for data management and coding. Thematic analysis using both inductive and deductive approaches, was employed to analyze the data. Initially transcripts were read multiple times to facilitate familiarization with the data. Open coding was conducted inductively, allowing codes to emerge from the participants’ narratives. The related codes were then grouped into categories or sub-themes, then subsequently the sub-themes grouped according to pre-determined themes based on the SEM as was used by Castillo-Neyra et al. (26).

Barriers were identified inductively from participants’ narratives and were defined as any factors that hindered effective dog bite case management or dog vaccination. Codes reflecting challenges or constraints or negative experiences were grouped into sub-themes and subsequently categorized as barriers. These barriers were then organized deductively according to the levels of the SEM, providing a structured framework. Finally, a codebook was developed including codes, sub-themes, themes and representative participant quotations (presented with their unique identifiers to ensure anonymity).

### Ethics approval and consent to participate

The study was approved by the School of Veterinary Medicine, Animal Resources Institutional Animal Care and Use Committee of Makerere University, Kampala, Uganda (Approval number: SVAR-IACUC/135/2023). Additionally, the study received approval by the Uganda National Council for Science and Technology (HS3463ES). Subsequently, the study received approval from the Soroti district local government health office. Research participants who were literate provided written informed consent in English, while those who were not literate, offered consent by using a thumbprint, in the presence of their preferred witnesses. Consent was sought after thorough explanation in the participants’ preferred language by a bilingual research assistant, of the purpose of the study, the benefits of the study, the participants’ rights to participate or withdraw from the study.

### Research team and reflexivity

At the time of the study, the research team comprised of individuals with diverse academic and professional backgrounds in medicine and public health. DoA and DiA were master’s students at Makerere University, while JK, TO, and SO were lecturers with doctoral-level training and experience in qualitative research. JK was a senior qualitative researcher, who provided methodological oversight throughout the study. TO, DoA and DiA had prior training in qualitative research before the start of data collection. None of the researchers had prior relationship with the study participants, which minimized potential influence on participant responses.

The team included both male (CK, TO, SO, and DiA) and female (JK, SD, SH and DoA), allowing for diversity in perspectives during data collection and analysis. CK, SD, and SH were part of a larger study focused on rabies elimination in Uganda. Regular debriefing sessions were conducted among the research team to reflect on evolving findings and to minimize individual researcher bias during data collection and analysis.

## Results

A total of 104 participants were included, with a fairly even distribution between Kamuda sub-county (52.9%) and Soroti municipality (47.1%). Focus group discussions comprised most participants (92.3%) and predominantly male (69.2%). The majority were aged 30–39 years (66.3%) and engaged in peasant farming (74.0%). Over half had attained secondary education (53.8%). Most respondents reported having a dog in the household (72.1%), although only 27.9% personally owned dogs as indicated in Table 1.

**Table 1:** Participants demographic information.

Variable	Category	Frequency (*n*)	Percentage (%)
Study site	Kamuda sub-county	55	52.9
	Soroti municipality	49	47.1
Participant group	FGD participants	96	92.3
	KII participants	8	7.7
Sex	Male	72	69.2
	Female	32	30.8
Age group	20–29	27	26.0
	30–39	69	66.3
	40–49	8	7.7
Occupation	Peasant farmers	77	74.0
	Religious leader	2	1.9
	Local leader	2	1.9
	Teacher	4	3.8
	Health worker	1	1.0
	Veterinary officer	1	1.0
	Other	17	16.3
Education level	No formal education	10	9.6
	Primary	29	27.9
	Secondary	56	53.8
	Tertiary	9	8.7
Dog ownership status	Dog owner	29	27.9
	Have a dog in household	75	72.1

**Figure 2:**
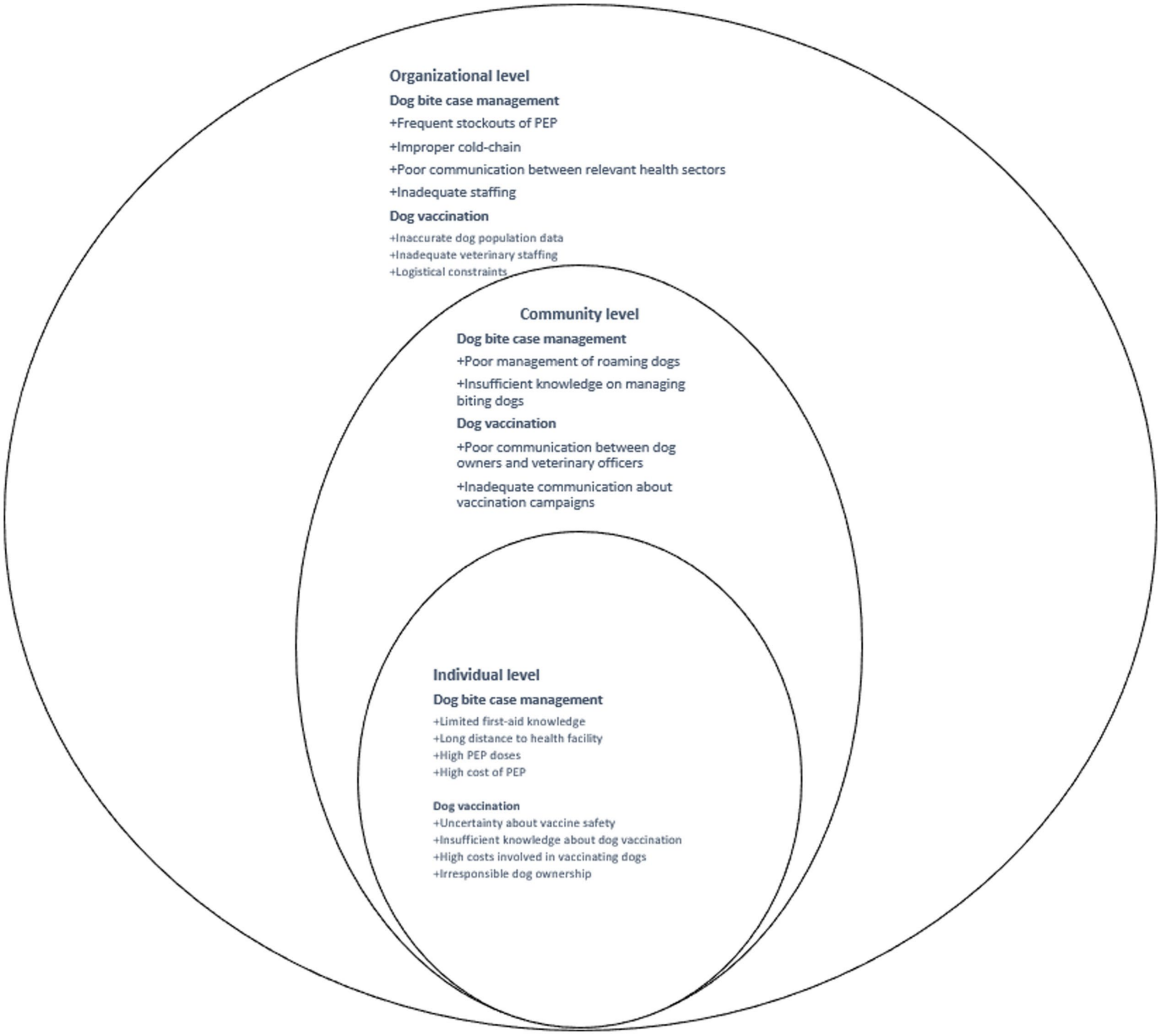
Modified socio-ecological model illustrating barriers to dog bite management and dog vaccination in Soroti district, Uganda.

The interviews yielded vital information on barriers to dog bite case management and dog rabies vaccination that was organized based on the constructs of the socio-ecological model (SEM) (26) as presented in Table 2 and Figure 2. The data was presented based on two themes, i.e., barriers to dog bite management, and barriers to dog rabies vaccination, organized accordingly by individual, community and organizational levels, although several barriers cut across these levels and influenced both thematic areas.

**Table 2:** A summary of the barriers to dog bite case management and dog rabies vaccination in two study areas of Soroti district, Uganda.

Themes	Sub-themes	Barriers	Quotations
Barriers to dog bite case Management	Individual level	Limited first-aid knowledge Long distance to health facilities for PEP High PEP costs High PEP doses	“*All we do is taking the dog bite victim to the health facility; it is the nurse who tells us when and how many times to come back for another dose.*” (FGD participant at Site 9) “*The best we do is to cover the wound with a cloth on the dog bite area and maybe sometimes the victim has to shower and in the process, they clean their wound. Some people wish to wash but fear to dog bite marks will be removed.*” (FGD Participant at Site 4) “*We mostly take the victims to Atiris Hospital for PEP which is about 20 km away but if they do not have then we are referred to either Mbale district hospital (114 km) or Entebbe General Hospital (350 km).*” (FGD participant at Site 7) *“That vaccine at the hospital for rabies is UGX 50,000 per dose. That is a lot of money for most of us so the owner of the dog that has bitten has to sale his cows, goats or even land just to get that money*.” (FDG participant at Site 5) “*For my scenario, my dog bit a 13 year old child and luckily, it was not killed but we isolated it and after 14 days it was still okay up to now so I only made payment for the victim’s 2 doses only.*” (Female FGD participant at Site 2)
	Community level	Poor management of roaming dogs Insufficient knowledge on managing biting dogs	“*We as a community kill the dog before it bites another person. We do not know of any other way of handling the culprit dog so, we do what our fore-fathers used to do.*” (Male participant at Site 10) “*Since mob killing of dogs is inhumane, we try to observe protocol for managing the culprit dog but sometimes we reach the site when the dog has been killed. However, if we arrive in the area early, we prevent the mob killing and take necessary measures.*” (KII-1)
	Organizational level	Frequent PEP stock outs Improper cold chain Poor communication between relevant health sectors Inadequate staffing	“*The NMS often takes long to supply these PEP vaccines and so when there is a stock out, we refer these patients to other government facilities like Tororo, Mbale or Entebbe general hospitals.*” (KII-2) “*We always have the vaccine at all times.*” (KII-5) “*Majority of these small health centers do not have refrigerators or constant power supply to the refrigerators and therefore, PEP vaccines cannot be supplied to them however, they are informed of where to refer the dog bite victim in case they receive any.*” (KII-2) “*We have few veterinary staff within the district and therefore they are not readily available to all villages to curb the situation before the culprit dogs are stoned to death.*” (KII-1) “*Around here, we do not have any veterinary officer who can manage dogs nor do I have any of their contacts of any veterinary officer.*” (KII-6)
Barriers to Dog vaccination	Individual level	Uncertainty about vaccine safety Insufficient knowledge about dog vaccination High costs involved in vaccinating (transport and paying) dogs Irresponsible dog ownership	“*All we do is taking the dog bite victim to the health facility; it is the nurse who tells us when and how many times to come back for another dose.*” (FGD participant at Site 9) “*The best we do is to cover the wound with a cloth on the dog bite area and maybe sometimes the victim has to shower and in the process, they clean their wound. Some people wish to wash but fear to dog bite marks will be removed.*” (FGD Participant at Site 4) “*We mostly take the victims to Atiris Hospital for PEP which is about 20 km away but if they do not have then we are referred to either Mbale district hospital (114 km) or Entebbe General Hospital (350 km).*” (FGD participant at Site 7) *“That vaccine at the hospital for rabies is UGX 50,000 per dose. That is a lot of money for most of us so the owner of the dog that has bitten has to sale his cows, goats or even land just to get that money*.” (FDG participant at Site 5) “*For my scenario, my dog bit a 13 year old child and luckily, it was not killed but we isolated it and after 14 days it was still okay up to now so I only made payment for the victim’s 2 doses only.*” (Female FGD participant at Site 2)
	Community level	Poor communication between dog owners and veterinary officers Inadequate communication about vaccination campaigns	“*If we miss vaccinating the dog during the campaign, we all need to wait until the next one.*” FGD participant at site 5.
	Organizational level	Inaccurate dog population data. Inadequate veterinary staffing Logistical constraints	*“We capture information on the name of the dog owner, number of dogs and their fur color.*” (KII-2) “*The budget allocated to rabies vaccination campaigns is really small and insufficient yet the logistics and transport required are often costly as compared to what is provided.*” (KII-1)

### Barriers to dog bite management

#### Individual level

Participants in all the FGDs were aware that dog bite victims are supposed to be rushed to the health facility to receive the rabies PEP vaccine to prevent the victim from acquiring rabies disease. Participants reported that the community had fear for rabies termed as “*a deadly disease*,” affecting humans and therefore took all necessary measures to ensure the dog bite victim especially children (common victims) received PEP. However, individual knowledge gaps were evident regarding the PEP regimen, as commented on by one participant stating,

“*All we do is taking the dog bite victim to the health facility; it is the nurse who tells us when and how many times to come back for another dose.*” (FGD participant at Site 9)

At the individual level, there was limited knowledge of appropriate first aid practices. Most participants reported that they could not provide first aid to a dog-bite victim. Only a few participants in some of the FGDs were aware of some steps in providing first aid to a dog-bite victim. These mentioned to either cover the wound with a cloth to prevent bleeding or cleaned it while showering before the victim was rushed to the health facility. However, others avoided cleaning the wound with fear of washing away the bite marks that were evidence of the victim being bitten by a dog.

“*The best we do is to cover the wound with a cloth on the dog bite area and maybe sometimes the victim has to shower and, in the process, they clean their wound. Some people wish to wash but fear to erase dog bite marks will be removed.*" (FGD Participant at Site 4)

These gaps in first aid knowledge were often reinforced by limited access to accurate information at community level and weak communication from health systems, liking individual practices to broader structural constraints.

Individual access barriers were also commonly reported especially in the rural areas. Long distances to the health facilities with PEP was a barrier to dog bite management, as they stated that bite victims had to move for distances ranging from approximately 20 to 350 km to receive PEP, as many of the nearby health facilities barely had the rabies PEP available.

“*We mostly take the victims to Atiris Hospital for PEP which is about 20km away but if they don’t have then we are referred to either Mbale district hospital (114km) or Entebbe General Hospital (350km).*” (FGD participant at Site 7)

Financial constraints at the individual or household level were a major barrier. Participants across both the KIIs and FGDs reported that the cost of rabies PEP was very high, making it difficult for dog owners to afford treatment for bite victims. Many noted that dog owners often became frustrated when they were forced to sell livestock or other property to cover treatment expenses. One participant explained,

*“That vaccine at the hospital for rabies is UGX 50,000 per dose. That is a lot of money for most of us so the owner of the dog that has bitten has to sale his cows, goats or even land just to get that money*.” (FDG participant at Site 5)

These financial challenges contributed to individual decisions to discontinue treatment, especially when symptoms subsided or when the biting dog appeared healthy, and were further compounded by long distances and frequent stock outs of PEP at nearby health facilities.

One participant shared,

“*For my scenario, my dog bit a 13-year-old child and luckily, it was not killed but we isolated it and after 14 days it was still okay up to now so I only made payment for the victim’s 2 doses only.*” (Female FGD participant at Site 2)

#### Community level

At community level, participants described shared norms and practices related to dog ownership and management. They noted with concern that there was improper management of dog movements and dogs freely roamed. Especially from rural communities, participants expressed no need to confine their dogs, stating that confined dogs always became aggressive, but also, owners could not afford feeding them every day, and so they let the dogs roam to find what to eat on their own, a practice that increased the likelihood of dog bites and placed additional burden on already constrained health services.

All participants in FGDs from both peri-urban and rural areas did not know about proper management of bite victims and the biting dog. For the biting dog, participants mentioned that any members available at the time of the bite incidence were responsible for hunting it down and beating it to death, which had been the practice from years back. For-instance one participant stated that,

“*We as a community kill the dog before it bites another person. We don’t know of any other way of handling the culprit dog so, we do what our fore fathers used to do.*” (Male participant at Site 10)

This collective response often occurred before veterinary or public health authorities could intervene, limiting opportunities for proper rabies investigation and control, especially in settings where veterinary personnel were few or delayed in reaching communities.

Although this practice had long been in place, there had been efforts by some stakeholders to educate the public about management of dog bite incidents. During these sessions, participants were informed that in case the culprit dog showed any symptoms suggestive of rabies and lacked evidence of current vaccination, the dog would then be euthanized and brain samples obtained and taken to the National Animal Disease Diagnostics and Epidemiology Centre (NADDEC) located in Entebbe, for confirmation of rabies. In cases where the dog appeared asymptomatic, it would be put under isolation for 14 days to observe for onset of rabies symptoms. If no symptoms appeared during this period, the dog would be released to its owner, however, if symptoms developed, the animal would be euthanized and brain tissue collected for diagnostic confirmation.

“*Since mob killing of dogs is inhumane, we try to observe protocol for managing the culprit dog but sometimes we reach the site when the dog has been killed. However, if we arrive in the area early, we prevent the mob killing and take necessary measures.*” (KII-1)

#### Organizational level

Across both FGDs and KIIs, participants reported frequent stock-outs of PEP in healthcare facilities especially those owned by the government (also called public health facilities). Some KIs further verified that this occurrence was due to irregular/inconsistent supply of PEP by the National Medical Stores (NMS), a governmental agency responsible for supplying public healthcare facilities with medicines, vaccines and medical devices.

“*The NMS often takes long to supply these PEP vaccines and so when there is a stock out, we refer these patients to other government facilities like Tororo, Mbale or Entebbe general hospitals.*” (KII-2)

These frequent stock outs directly contributed to the long distances travelled by patients and increased out-of-pocket costs, reinforcing individual-level access barriers.

However, some key informants from a private facility reported constant supply of PEP doses at their facility since they often purchased doses on their own.

“*We always have the vaccine at all times.*” (KII-5)

Lack of a reliable cold chain system in many healthcare facilities especially level II, III and smaller private clinics, was mentioned by majority of KIs, and this made it difficult to store and provide PEP to victims.

“*Majority of these small health centers do not have refrigerators or constant power supply to the refrigerators and therefore, PEP vaccines cannot be supplied to them however, they are informed of where to refer the dog bite victim in case they receive any.*” (KII-2)

Participants also highlighted inadequate veterinary staffing as a key challenge. With too few veterinary personnel available across the district, biting dogs were often mishandled by community members, including being stoned to death before proper assessment could be made.

“*We have few veterinary staff within the district and therefore they are not readily available to all villages to curb the situation before the culprit dogs are stoned to death.*” (KII-1)

This limited workforce not only affected timely response to bite incidents but also contributed to community practices such as mob killing of dogs due to absence of professional guidance.

The interviews further revealed minimal communication and coordination across the veterinary, human health sector and the local government. This lack of proper linkage often left communities unsure about where or how to seek care following dog-bite incidents. Veterinary officers frequently referred victims to health facilities without knowing whether PEP was available, and therewere no established communication channels across the sectors.

“*Around here, we do not have any veterinary officer who can manage dogs nor do I have any of their contacts of any veterinary officer.*” (KII-6)

This poor coordination further contributed to knowledge gaps at individual level and uncertainty within communities on how to appropriately respond to dog bite incidents.

### Barriers in dog vaccination

#### Individual level

All participants in FGDs were aware that dogs required vaccination to prevent rabies.

“*Most of us try to vaccinate our dogs because each time they announce a rabies vaccination campaign, we take our dogs and cats.*” (FGD participant at Site 5)

However, some participants were unsure about the recommended vaccination schedule and relied entirely on government announcements before vaccinating their animals.

“*We wait until they announce for rabies mass vaccination in order to vaccinate our dogs.*” (FGD participant at Site 2)

This reliance on announcements reflects how individual practices are shaped by availability and effectiveness of communication from veterinary services.

Only a few participants, mainly from Soroti municipality reported vaccinating their dogs annually, even when there was no mass campaign. They contacted veterinary officers directly for vaccination or treatment, in absence of formal mass campaign.

“*Some of us have contacts for a veterinary doctor that we call every year to vaccinate, deworm or incase the dog is sick.*” (FGD participant at Site 1)

Individual participants especially from Kamuda sub-county reported lack of proper communication about the rabies vaccination campaigns. Some participants mentioned that they found out about the vaccination campaign on the very day it was happening and either they rushed home to get their dogs or missed it. Others reported missing the campaign because they were away for work or traveled and there was no one to take their dogs for vaccination.

Cost was another cited barrier. Some participants reported that sometimes they did not take their dogs for vaccination because the cost of vaccination was high especially for people that had more than one dog.

“*The cost of vaccinating each dog is UGX 5000 so if someone has five dogs, that becomes too much yet most of us to not have that money.*” (FGD participant at site 10)

Participants also reported weak or non-existent relationships with veterinary officers. For-example, participants from communities in sites 7 and 10 in Kamuda sub-county were unaware of any veterinary professional capable of managing dog health concerns. In some areas however, like in site 1 in Soroti city, most participants had a contact to either a veterinary officer or animal husbandry officer who could treat and even vaccinate their dogs at any desirable time and date. Such appointments would be set either individually or as a community, though, this would come at a higher cost.

Transporting dogs to designated vaccination points was another reported challenge. Individuals described their dogs as aggressive, and this was even worse for people with more than one dog. This was further complicated by the long distances that they had to move to vaccinate their dogs, given the vaccination exercise was often at static points, as explained by one participant stating that,

“*Vaccination campaigns are usually set up at one point in the parish so all villages within that parish receive rabies vaccination at the same point. This makes distances to be traveled longer.”* (KII-3)

While majority of dog owners considered their dogs to be members of the family, participants pointed out that some dog owners only kept dogs for security but did not care for them, and others often abandoned their dogs whenever they relocated to different places. One participant explained that,

“*There are people that had dogs but relocated to a different area and left the dog behind, so those dogs now become stray dogs and move, eat and reproduce anyhow and you find that they are the ones that bring these diseases into our community*.” (FGD participant at Site 3)

Participants reported with concern about ownerless dogs, that no one was prepared to take un-owned dogs in the community for rabies vaccination. They added that there were some dog owners who are “careless” and lacked motivation to invest their money, time and effort on their dogs.

“*Even when they are aware of an ongoing vaccination, they claim that they can’t waste money on a dog that does not bring back money in their pocket.*” (KII-4)

These practices by contributed to a persistent pool of unvaccinated dogs, linking individual behavior with gaps in enforcement and resource allocation.

Some other dog owners claimed that their dogs were at low risk of acquiring rabies since the disease was rare in their area.

#### Community level

The majority of FGD participants except those at Site 1, reported not being aware of the different channels to get their dogs vaccinated. Most participants believed they had to wait for mass vaccination campaigns to get their dogs vaccinated and were unaware that they could contact a veterinary officer directly at any time. This limited awareness of alternative vaccination pathways reflected broader communication gaps within the system.

“*If we miss vaccinating the dog during the campaign, we all need to wait until the next one*” FGD participant at site 5

Participants also reported poor communication regarding the dates for mass vaccination campaigns. In an interview with KII-6 from Awiniyi village, he explained that communication from the sub-county offices was shared with the parishes and then to the villages, specifically to the chairpersons. The respective village chairpersons then announced the information to the community members through megaphones, community radios and community gathering like in churches. Although this was a correct channel, participants noted challenges with timing. Some received the information only a few days before the campaign, on the day of vaccination, or not at all, resulting in missed opportunities to vaccinate their dogs.

#### Organizational level

There was insufficient information regarding dog population, i.e., the number of dogs in the district, as there were no studies done to make this estimation. Information captured during vaccination was insufficient to give this detail, but also, vaccination was not done for the entire district every time it occurred, leading to missing of data from dog owners who did not present their dogs for vaccination.

*“We capture information on the name of the dog owner, number of dogs and their fur color.*” (KII-2)

This lack of data contributed to under-supply of vaccines, which in turn affected access at community and individual levels.

Participants added that this insufficient information on dog population estimates has contributed to the district being allocated fewer vaccines than are required to vaccinate all dogs in the district.

Another notable barrier as stated by key informants was the inadequate number of veterinary officers to conduct mass vaccination campaigns, which would mean more time for the available human resource to cover all communities for vaccination. This constraint affected both vaccination coverage and response to dog bite incidents, demonstrating its cross-cutting nature.

Additionally, inadequate logistics and transport means to conduct mass vaccination in all communities was reported by one of the key informants as the budget allocated to rabies vaccination campaigns was always smaller than the expenditure required ensuring vaccination of dogs in all communities. This limitation further interacted with distance and cost barriers experienced by individuals and communities.

“*The budget allocated to rabies vaccination campaigns is really small and insufficient yet the logistics and transport required are often costly as compared to what is provided.*” (KII-1)

These organizational challenges collectively limit the reach and effectiveness of rabies vaccination campaigns in the district, with several barriers, particularly financial constraints, communication gaps and limited veterinary capacity, cutting across levels and influencing both prevention and case management pathways.

## Discussion

This study revealed multiple barriers to dog bite case management and dog rabies vaccination at individual, community and organizational levels of the SEM. Two themes, i.e., barriers to dog bite case management and barriers to dog rabies vaccination, were pre-determined, and findings were presented based on these themes. These findings collectively indicate persistent gaps that affect progress toward the global goal of eliminating human rabies deaths by 2030 particularly in resource limited settings such as Soroti.

The demographic profile of participants provides critical context for interpreting these findings. The predominance of peasant farmers indicates that most households operate within subsistence-based economic systems, which likely amplifies the financial barriers reported, including the cost of PEP and dog vaccination. Similar patterns have been observed in rabies-endemic regions where low-income rural households face substantial challenges in accessing and completing PEP due to financial constraints (2, 40). Additionally, the concentration of participants within the economically active age group (30–39 years) suggests that indirect costs, such as time away from income-generating activities, may further delay or limit care-seeking (41). The predominance of male participants may also have influenced perspectives on dog ownership and decision making, which are often gendered in rural African settings (42).

### Barriers to dog bite case management

The barriers to dog bite case management included limited knowledge about first aid, long distances to facilities for PEP, high PEP doses, high cost of PEP, poor management of roaming dogs, insufficient knowledge on managing biting dogs, frequent PEP stock outs, improper cold-chain, poor communication between relevant health sectors, and inadequate staffing.

When a dog bites a person, basic first aid should be provided to the bite victim before rushing them to the healthcare facility (43). This involves washing the wound with soap and water for at least 15 min to reduce rabies virus transmission (44). In Soroti, most participants lacked appropriate knowledge regarding provision of first aid to a bite victim, partly due to limited outreach, lack of local health education campaigns, and inadequate staffing at health facilities. Some participants mentioned that first aid was not provided because of fear that the bite marks would be washed away, fearing that the dog bite victim would not be considered eligible for PEP. Some participants reported offering some form of assistance to bite victims without being aware that their actions constituted basic first aid. This was a similar finding in a related study conducted in Ethiopia by Kabeta et al. (18), which similarly reported low levels of awareness and inappropriate first aid practices among dog bite victims and their caretakers. These gaps illustrate how organizational-level constraints directly influence individual-level knowledge and practices.

Furthermore, most respondents in this study were not aware of the recommended number of PEP doses that a dog bite victim had to receive and only followed recommendations from a healthcare worker at a facility. This gap is linked to insufficient follow-up mechanisms, limited staffing levels, inadequate community-level education as identified in our study. This illustrates how organizational-level barriers exacerbate individual-level challenges. Similar findings have been reported in studies from Ethiopia and Kenya, where many victims receiving PEP were not aware of how frequent they had to come back for subsequent PEP doses and the importance of completing the full vaccination schedule (18, 45). Such knowledge gaps highlight the need for targeted health education/sensitization interventions to improve awareness of appropriate PEP regimens and adherence to vaccination schedules.

Despite the community’s effort to ensure that dog bite victims received PEP, long distances to reach healthcare facilities was flagged as a key barrier. For instance, participants during interviews reported that bite victims accessed PEP at healthcare facilities located 20–350 km away from their homes, which was very costly and quite discouraging completion of the regimen. The long distances reflect limited decentralization of PEP services in Soroti and resource constraints that prevent establishing more peripheral access points. In addition, limited supply from National Medical Stores, or absence of reliable cold chain system especially at peripheral healthcare facilities to maintain the PEP viable compound this situation, leading to high out-of-pocket costs, reluctance to initiate or complete the regimen, and other cyclical barriers to effective dog bite management in Soroti. Previous studies in Africa have documented similar challenges such as distance travelled and cost of transport or treatment regimen (19, 20, 25, 30). In Tanzania, the decentralized and free PEP provision significantly improved access, whereas long distance and vaccine shortages were linked to non-completion (19). These findings highlight the need for strengthening supply chains, ensuring PEP is available closer to high-risk communities and reducing financial and geographical barriers which are critical for improving dog bite management.

Closely linked to geographical barriers, the high cost of PEP reported in this study further constrains timely initiation and completion of treatment regimen, thereby increasing the risk of preventable deaths. Financial barriers to PEP access have been reported in previous studies and remain critical bottle bottleneck to achieving the “Zero by 30” target (19, 46). In Tanzania, unaffordable direct and indirect costs including travel, were associated with delayed initiation, abandonment and non-completion of PEP, contributing to preventable deaths (19, 47). Emerging global efforts including from Gavi, the vaccine alliance, aim to expand equitable access to human vaccines and strengthen delivery systems, which could address the affordability challenges such as those identified in this study (48).

The study also revealed poor intersectoral coordination between veterinary and human health sectors, creating gaps in communication on the availability of PEP and proper management of bite victims. Veterinary staff often referred victims to health care facilities without knowing whether PEP was available, while human healthcare workers lacked information about the health or vaccination status of the biting dog. This situation in Soroti arises from limited formal communication channels, data sharing systems between sectors, thus reflecting systemic challenges within the One Health framework, where fragmented coordination limits effective rabies control as revealed from other studies (31).

Participants reported handling the biting dogs through mob killing immediately a bite incident occurred. This situation denied veterinary staff an opportunity to access a biting dog, and this was a widespread practice. While such actions stem from fear and lack of awareness, they hinder proper observation and testing of the suspected rabid dog. Similar practices have been reported in Tanzania and Nigeria, where communities frequently kill suspected dogs instead of observing them for 10–14 days as per WHO and OIE guidelines (43, 49). Kimera and Mlangwa (50) stated that policies to prevent rabies through killing of stray domestic dogs and feral dogs and cats exist in some developing countries, however, recent research has shown this method to be ineffective in eliminating rabies but still this option remains in place in some countries worldwide. Such findings underscore the urgent need for community sensitization on humane handling and observation of biting dogs, in line with established One Health and animal welfare protocols.

Such practices, although driven by fear and limited awareness, have key implications for rabies control. Mass dog culling has been shown to be ineffective and may limit rabies control efforts, reducing vaccination coverage and community trust (51). Immediate killing of suspected dogs prevents the recommended observation time and limits opportunities for laboratory confirmation, thereby weakening surveillance systems. This disrupts coordinated One health responses and may obscure the true burden of disease, thus limiting progress toward Zero by 30 strategy (52, 53). Strengthening community awareness alongside veterinary response capacity is therefore critical to discourage such a practice.

### Barriers to dog rabies vaccination

Dogs are the main transmitters of rabies to the human population and the most effective strategy of eliminating the disease is through vaccination of dog (35). However, the study identified barriers to dog rabies vaccination at an individual, community and organizational level which result to persistence of rabies in communities having dogs. These barriers included inadequate knowledge on frequency of vaccination and access points for vaccination services, poor communication, aggressive and unrestrained dogs, insufficient veterinary staff, inadequate supply and cold chain system, long distances to vaccination sites and high associated costs.

While many participants demonstrated considerable awareness regarding taking their dogs for vaccination to prevent them from catching rabies, few knew how often vaccination should occur and where or how the service should be. Additionally, they did not know, especially in rural communities that one could invite the veterinary officer whenever they needed to vaccinate or treat their dog. This was a similar finding in Kenya, where a related study reported that only 2% of respondents knew that veterinary staff vaccinate animals, 11.2% had never heard of any veterinarian and 42.3% were unsure they existed (22). During our study, some dog owners reported to sit back waiting until the district announced mass dog vaccination. This was probably due to reported limited veterinary capacity to enable organization of routine campaign outreaches. Similar dependency on centrally organized vaccination drives had been reported in other studies in Uganda, Tanzania and Ethiopia (49, 54, 55).

Communication regarding dog vaccination campaigns were often announced only days before commencement of the campaign itself. However, findings indicate that some dog owners were either receiving information late or not receiving it at all, as similarly observed in a study in Peru (36). This poor communication may result from limited staffing at veterinary offices, lack of systematic outreach planning and competing priorities. Additionally, delayed information can reduce community trust in official messages if prior campaigns were inconsistent. This results in low participation in vaccination within the community and thus realizing the recommended 70% coverage to obtain herd immunity becomes challenging (11). In our study, majority of participants pointed to inadequate communication about dog vaccination campaigns as the main contributing factor for non-participation. Although one key informant from Awiniyi village clearly explained how the communication regarding the campaigns flowed which was reasonable, several dog owners during FGDs mentioned that quite often information about vaccination reached them on the very last day, and late announcements competed with other priorities which caused them to miss participation, a pattern observed elsewhere. As stated by other researchers, delayed or poorly advertised campaigns have been associated with low turn up in Uganda and Tanzania, and better community engagement have shown to improve participation (55–57). Strengthening community-level communication and coordination is crucial for improving vaccination coverage.

Free-roaming and ownerless dogs were another major challenge. As in other communities in Uganda especially in rural areas, peri-urban and slum areas in urban areas (25, 28), this study found that majority of dog owners let their dogs roam freely in search for food and mates. Some dog owners did not care and completely abandoned their dogs. Soroti district being an agricultural and pastoral area, receives seasonal migrants from other districts who come and practice farming. These contribute to high numbers of unvaccinated dogs as community members reported that whenever households relocated either back home or to other areas, they left their dogs behind without arranging for adoption by a new owner. Consequently, these dogs were excluded from vaccination programs, including free campaigns. Such situations are not limited to Soroti district. Generally across Uganda, especially in pastoral and agricultural areas, free-roaming and ownerless dogs remain a persistent challenge to achieving adequate vaccination coverage (55, 57). This demonstrates how inaccurate dog population data at the organizational levels contributes to community-level challenges in managing roaming dogs. Moreover, participants reported that some dog owners do not consider their dogs to be part of the family. These dog owners were described as “careless or adamant”, as they were unwilling to have their dogs vaccinated, perceiving them as unworthy of protection or unlikely to get infected. They are not willing to spend their money on a dog they consider having low value (20). Similar attitudes, including laziness, irresponsibility and lack of concern for dogs have been reported in other communities (19, 20, 24, 30, 58). This hampers the achievement of herd immunity against rabies in the community and highlights the need for targeted community awareness and enforcement of responsible dog ownership.

While most respondents expressed willingness to contribute financially to rabies vaccination at a low cost, others, particularly in rural areas faced financial constraints hence their appeal for free vaccination from government. These dog owners earn meagre incomes that barely sustain their own needs, making it difficult for them to afford dog vaccinations while others do not see the financial benefit of it. This issue of poverty as a barrier to rabies elimination has been highlighted in other studies as well (28, 39). Currently, the government in Uganda supplies vaccines to the districts at no cost (15), but due to the logistics involved in mass vaccination campaigns, the local government offer rabies vaccination services at a subsidized cost. However, respondents still perceived the price to be high and expressed a willingness to opt for either not to vaccinate or go for free vaccination whenever this happened, even if it meant traveling to distant locations, this aligns with findings from a relayed study conducted in Tanzania (40).

Long distances to the vaccination sites compounded by the difficulty of handling aggressive dogs during transport, was identified as key barrier to achieving adequate dog vaccination. This would be mitigated through using alternative vaccination strategies such as door to door, integrated dog and livestock vaccination, integrated dog vaccination with human health services, and school-based approaches (9, 56, 57). The different alternatives provide dog owners with more convenient options. Door to door vaccination can bring services directly to households, while integrated strategies allow owners to access multiple services simultaneously. These findings are consistent with pilot vaccination studies in Uganda, which demonstrated that integrated and community based approaches significantly improve participation and coverage, particularly for dogs that are difficult to hand or transport (57).

Participants in this study expressed concerns regarding inadequate availability of information regarding dog population. Although the government provides vaccines for free to the districts, provision of adequate doses depends on accurate estimates of dog population. Similarly, district level budgeting for vaccination campaigns is guided by projected dog population sizes. In the absence of these estimates in Soroti, the veterinary sector often receives insufficient vaccine quantities. This challenge has been documented in other settings. Lack of accurate dog census has constrained strategic planning, leads to underestimation of vaccine requirements, and ultimately hinder progress toward achieving a high vaccination coverage threshold for herd immunity (2, 9, 27). The lack of information on dog population, maybe caused by lack of systematic dog registration, insufficient community engagement, and resource constraints at district level.

Addressing these barriers requires not only local system strengthening but also leveraging global support mechanisms. For example, access to affordable and quality-assured dog vaccines remains essentials for achieving the 70% recommended vaccination coverage needed to interrupt transmission (43). Initiatives such as by WOAH vaccine banks provide countries with a reliable mechanism to procure rabies vaccines at reduced costs, which potentially could help overcome logistical and financial burden identified in this study (59). Integrating such mechanism into national rabies control programs could significantly accelerate progress to Zero by 30 global strategy.

Dog vaccination, provision of PEP, and community education are key interventions outlines in the National Rabies Elimination Strategy of Uganda (60), aligned with the global “Zero by 30” target. Barriers effecting these interventions, as identified in this study, threaten progress toward this goal and, if unaddressed, may significantly hinder rabies elimination efforts.

Routine dog rabies vaccination and proper dog bite management are crucial to ensuring the safety of both human and animal health through prevention and control of rabies. It was interesting to note that dog owners only utilized rabies vaccination and PEP (accessed at healthcare facilities) as modern remedies for preventing rabies and managing dog bite victims. Notably, neither rural nor peri-urban dog owners reported beliefs or misconceptions that discouraged them from vaccinating their dogs or seeking PEP. Beliefs or misconceptions refer to traditional practices, cultural norms or misinformation that could lead owners to avoid vaccination or rely on ineffective remedies. Contrary to these findings, a study conducted in Masaka, Uganda, reported that some dog owners resorted to using herbal medicine for rabies prevention and treatment in dogs and humans (25, 47). Similarly, in western Uganda, some dog owners believed that the vaccine was harmful to dogs and instead used traditional remedies for rabies management (5). These discrepancies highlight the need for localised behavioural and social research to guide risk communication and community engagement strategies for rabies control and eventual elimination.

The study had some limitations. First, data were collected from a few selected rural and peri-urban communities, which limit the generalizability of the findings to other districts or settings with different socio-economic or cultural contexts. Second, the study relied solely on qualitative methods; while this provided rich, in-depth insights, it may have excluded perspectives that could have been strengthened or quantified through complementary quantitative approaches. A subsequent exploratory survey is therefore recommended to compliment the findings of this study.

### Conclusions and recommendations

The key challenges to dog bite management and dog rabies vaccination included limited knowledge of appropriate first aid and PEP regimens among dog bite victims, reliance on mass vaccination campaigns rather than routine vaccination, free-roaming and aggressive dogs, poor communication about campaign schedules, mob killing of biting dogs, insufficient veterinary staff, inadequate logistics, PEP stock outs, lack of accurate dog population data, and limited cold chain system. Despite these barriers, the findings also revealed high community awareness of rabies risk and willingness to participate in preventive measures, representing an opportunity to strengthen rabies control efforts.

To address these barriers, the district human and veterinary health authorities should implement sustained community education programs on dog bite first aid, timely seeking of care, and vaccination schedules. Communication strategies should e strengthened through the use of local leaders, media platforms, and community networks to ensure timely dissemination of vaccination campaign information. Access to dog vaccination should be expanded through integrated approaches such as through door to door, integrated campaigns and school-based programs. At organizational level, there is a need to strengthen capacity by increasing veterinary staffing, ensuring consistent availability of PEP and vaccines through improved supply chain management, and maintaining functional cold chain systems. Local government should also establish reliable dog registration and census systems to support effective vaccination planning. Additionally, promoting One Health collaboration between veterinary, human health and local government sectors can improve coordination in bite management, PEP distribution, and rabies surveillance. Implementing these strategies in a coordinated manner can increase vaccination coverage, improve dog bite management and advance progress toward eliminating human deaths from dog medicated rabies in Uganda. Aligning national efforts with global strategies such as Zero by 30, including leveraging support from Gavi and WOAH, maybe critical in addressing the interconnected financial, logistical and systemic barriers identified in this study.

## Data Availability

The data presented in the study are deposited here: https://zenodo.org/records/18679156.

## Appendix A1

### Data collection tools

Date: ……………………………

Dear correspondent, this exercise will be treated with highest level of confidentiality and your cooperation will be appreciated.

Objective 3: To understand the barriers to dog bite case management and dog vaccination in Soroti district.

This study does not have direct benefit to you, but the information obtained will be relevant for developing tools relevant in supporting rabies elimination in Soroti district and Uganda as a whole.

### Key informant interview guide

*Study title*: Barriers to proper dog bite case management and dog rabies vaccination in Soroti district, Uganda.

1. Based on your experience, what do dog owners in this community know about rabies, its prevention and rabies vaccination for dogs?
2. How is rabies vaccination of dogs generally approached and practiced in this community, including frequency and access to vaccination services?
3. What typically happens when a dog bites a person in this community, including actions taken by the dog owner, care sought by the victim, and management of the biting dog?
4. To what extent are dog bite incidents reported to authorities or veterinary personel in this community?
5. What factors prevent or discourage dog owners from vaccinating their dogs against rabies?
6. What challenges do dog owners face in accessing rabies vaccination services? Beliefs, norms, logistics etc.?
7. What measures do you think would improve rabies vaccination coverage in this community?

### FGD with dog owners

*Study title*: Barriers to proper dog bite case management and dog rabies vaccination in Soroti district.

Location: County: ………………………… Village: …………………………….

1. Can someone tell us the value of dogs in their households and community?
2. What do people in this community know about rabies? Transmission, signs, severity etc.
3. What is the general practice of dog vaccination in this community, including where vaccination services are obtained and how often?
4. What factors encourage or discourage dog owners from vaccinating their dogs against rabies in this community?
5. Can you describe what exactly happens when a person is bitten by a dog in this area?
6. What challenges prevent proper dog bite case management in this community, including medical care, reporting of cases and managing bite victims.
